# Evaluation of the Hybrid Membrane of ZnO Particles Supported in Cellulose Acetate for the Removal of Lead

**DOI:** 10.3390/membranes13020123

**Published:** 2023-01-18

**Authors:** Irma Pérez-Silva, Ma. Elena Páez-Hernández, Israel S. Ibarra, Rosa Luz Camacho-Mendoza

**Affiliations:** Área Académica de Química, Universidad Autónoma del Estado de Hidalgo, Carr. Pachuca-Tulancingo Km. 4.5, Mineral de la Reforma, Pachuca 42184, Hidalgo, Mexico

**Keywords:** hybrid membrane, ZnO, lead, battery industry

## Abstract

Water polluted by discarded heavy metals such as lead is creating a global pollution problem. In this work, adsorption of Pb(II) was realized in batch studies by a hybrid membrane of cellulose acetate with ZnO particles. First, ZnO particles were prepared by precipitation and immobilized on the membrane. The hybrid membrane was elaborated by interfacial polymerization. The structure and surface were characterized based on Fourier-transform infrared spectroscopy (FTIR), thermogravimetric analysis (TGA), and scanning electron microscopy (SEM). Batch experiments were carried out under different conditions where the number of particles of ZnO present in the membrane and the pH of the aqueous solution were varied. The Langmuir and Freundlich isotherm models were evaluated in the best adsorption conditions. Data fitted well with a Langmuir model with a maximum adsorption capacity of 15.55 mg·g^−1^, which was similar for this type of materials. Thermodynamic parameters such as Gibbs free energy, enthalpy, and entropy showed that the process was spontaneous and favorable. The hybrid membrane was evaluated in simulated wastewater of the battery industry with a superior efficiency of up to 97%; without the medium, it did not generate interference. These results suggest that Pb(II) removal by hybrid membrane is possible.

## 1. Introduction

Today, many water bodies have been contaminated due to the presence of various chemical compounds. Factors that contribute to water pollution include pesticides, fertilizers, industrial and urban waste, and heavy metals [1].

Heavy metals are bioaccumulated, biomagnified, and toxic in small amounts. For these reasons, and to avoid diseases and ecosystem damage, it is necessary to remove them from water [2]. Different treatments for removing heavy metals have been developed, such as precipitation, ion exchange, electrochemical techniques, and solvent extraction. However, these have low efficiency and high energy consumption and provide incomplete removal [3]. Lately, membrane technology has been used for the secondary and tertiary municipal treatment of wastewater, and, in other cases, one membrane process has been used for producing water of increasing purity and quality for various purposes [4].

Some types of membranes have been developed for this purpose, for example, organic, inorganic, and hybrid membranes. Hybrid membranes are a mix of organic and inorganic membranes, combining their basic properties for obtaining morphological stability, high selectivity and flux, a good thermal and chemical resistance, and an appropriate ratio between hydrophilicity and hydrophobicity [5,6]. Hydrophilicity and ion exchange capacity in the membrane are caused by different inorganic compounds, such as Ag, TiO_2_, ZnO, CuO, carbon nanotubes, graphene oxide, Al_2_O_3_, SiO_2_, Fe_3_O_4_, ZrO_2_, active groups, and zeolite, which are entrapped into the polymer film by track etching, stretching, sintering electrospinning, interfacial polymerization, dip coating, pressurized deposition, or phase inversion technique [7,8,9,10]. This can contribute to solving the different problems connected to each of them and increase the ability of membrane systems in different fields of the same energy sector and water treatment [11]. These membranes can be considered a new generation of membranes for inhibiting microbes, and eliminating charged pollutants such as heavy metals and dyes, and they have the possibility of being used on a pilot scale and at an industrial level [12]. For example, a hybrid membrane with graphite was used as an ultrafiltration membrane to remove Chrysophenine GX in textile wastewater [13]. A polyether sulfone modified with a graphene oxide membrane was used to treat tannery industry wastewater with a high rejection efficiency of chromium (˃99%) [14]. Other uses are the removal of antibiotics with a pillararene-MXene membrane [15] and the removal of dyes (77–100%), phenols (40–100%), and heavy metals (66–100%) [16]. For this reason, the present work focuses on the development and characterization of a hybrid membrane of acetate cellulose particles of ZnO. Lightness and pliability are provided by polymeric material [17]; in this case, the cellulose acetate is biodegradable, cheap, and an abundant organic matrix [18], while the particles of the oxide have the function of stabilizing the polymeric membrane, giving higher selectivity, antimicrobial activity, and better performance [19].

The evaluation was realized with a lead solution, which has been used since prehistoric times for the elaboration of pigments, electrical shielding, solder, glazes, and pesticides due to its properties such as ductility, malleability, conductivity, resistance to corrosion, and low melting point [20]; but, one of the main applications of lead is in storage batteries due to its special reversible reaction with sulfuric acid. Wastewater of this industry can contain between 3 and 15 mg·L^−1^ of soluble lead, which is becoming a source of pollution; there needs to be different alternatives for its treatment [21]. The factors evaluated were pH in feed phase, concentrations of the ZnO, contact time, adsorption isotherms, and thermodynamics parameters. Adsorption of lead in simulated water was also investigated.

## 2. Materials and Methods

### 2.1. Materials

Marjoram was acquired from the local market. N, N′-dimethylformamide (DMF), HCl 37%, and lead nitrate (Pb(NO_3_)_2_) were purchased from J. T. Baker (Xalostoc, Edo. Mex, Mexico; sodium hydroxide (NaOH, St. Louis, MO, USA) and cellulose acetate (AM. 50000, CA, Milwaukke, USA) were purchased from Aldrich. Zn(NO_3_)_2_·6H_2_O 99% was purchased from Fluka (Buchs, Switzerland). All the chemicals that were used were ACS grade. For the quantification of lead and simulated wastewater, solution standards for inductively coupled plasma and direct-current plasma (ICP/ DCP) from Fluka (St. Louis, MO, USA) were used. Aqueous solutions were prepared by dissolving the respective analytical grade reagent in deionized water with a resistivity of no less than 18.2 MΩ cm obtained by a Milli-Q Plus system (Millipore, Bedford, MA, USA).

### 2.2. Methods

#### 2.2.1. Preparation of Particles of ZnO

First, an extract was prepared, and 1.3 g of marjoram was put into 15 mL of deionized water in a heater at 50 °C for 30 min; the extract was cooled at room temperature and filtered for further studies. For the preparation of ZnO particles (NP), 2.7 g of Zn(NO_3_)_2_·6H_2_O was stirred with 10 mL of deionized water for 30 min. A 1 mL amount of extract and NaOH 2M were added into the solution until pH reached 12 and stirred at room temperature for 2 h. NP were washed and separated by centrifugation until excess base was removed (pH < 8). After, the product was dried at 60 °C for 12 h according to the report by Mohammadian et al. [22].

#### 2.2.2. Preparation of Hybrid Membrane

Polymeric solution was prepared by dissolving CA in DMF (10% *w*/*v*) after an amount of NP was added to the polymer solution and stirred for 30 min. The polymeric solution was poured onto a glass plate (thickness ~0.36 mm) and formed the hybrid membrane (CA/ZnO) by phase inversion employing water [23].

The morphology of the hybrid membrane (CA/ZnO) was examined by scanning electron microscopy (SEM) (JEOL JSM-6300, Tokyo, Japan). The membrane samples were mounted with conductive glue to metal stubs with the fractured edge up and then coated with gold sputtering. The estimate of immobilized zinc atomic percentage was determined by energy-dispersive X-ray spectroscopy (EDX) using a SEM JEOL JSM-5600 LV model.

Stability of the membrane was evaluated by thermal gravimetric analysis (TGA) with a Mettler Toledo TGA/SDTA 851e (Switzerland) in a temperature range of 50–800 °C and at a heating rate of 10 °C/min. Infrared analysis was carried out with a Perkin Elmer System 2000 with Fourier transform (Waltham, MA, USA).

Porosity percentage of the hybrid membrane was measured according to the report of Yang et al. (Equation (1)). A 2 cm^2^ amount of hybrid membrane was put into contact in deionized water at 24 h after the membrane was weighed and after its surface water was absorbed by filter paper (*W_W_*). The wet membrane was dried in an oven at 60 °C for 12 h before it was weighed (*W_d_*).

(1) $$Pr=\frac{W_{w}-W_{d}}{Sd\delta_{w}}\times 100$$

where *d* is the average thickness of the membrane and *δw* the density of water to room temperature [23].

An approximation of the contact angles of membranes was measured with a digital microscope. A water drop (10 μL) was put onto different points on the membrane’s surface to determine static contact angles.

#### 2.2.3. Sorption Procedure

To evaluate the adsorption percentage of the membrane ($\%A_{Pb}$) (Equation (2)), 10 mL of 10 mg·L^−1^ of lead was put in contact with a superficial area of 5 cm^2^ of CA/ZnO for 4 h in batch. All the experiments were performed three times, while the amount of lead was measured in a flame atomic absorption spectrometer VARIAN SpectrAA-880 (Australia).

(2) $$\%A_{Pb}=\left(\frac{C_{0}-C_{f}}{C_{0}}\right)100$$

Adsorption isotherm experiments were realized by putting 0.06 g of CA/ZnO 3% (*w*/*v*) in contact with 10 mL of a solution of Pb(II) in the range of 40–800 mg·L^−1^ at room temperature. Langmuir and Freundlich linear forms were used to model the adsorption (Equations (3) and (4), respectively) [24].

(3) $$\frac{C_{e}}{q_{e}}=\frac{1}{q_{max}K_{L}}+\frac{C_{e}}{q_{max}}$$

(4) $$logq_{e}=logK_{f}+\frac{1}{n}logC_{e}$$

where *q_e_* = amount adsorbed (mg.g^−1^), *C_e_* = equilibrium concentration of adsorbate (mg.L^−1^), *q_max_* = maximum adsorption capacity (mg·g^−1^), *K_L_* = Langmuir constant of adsorption, *K_f_* = adsorption capacity (mg·g^−1^), and *n* = adsorption intensity.

Both models permitted us to describe whether the adsorption was in a homogeneous form (Langmuir) or a heterogeneous surface (Freundlich). The error function for evaluating the fit of the isotherm was evaluated by chi-square test (Equation (5)) [25].

(5) $$\chi^{2}=\sum^{\;}\frac{{\left(q_{ex}-q_{e,m}\right)}^{2}}{q_{e,m}}$$

where *q_e,m_* is the equilibrium capacity (mg·g^−1^) obtained from the model, and *q_ex_* is the experimental data of the capacity after the sorption process (mg·g^−1^).

Thermodynamic studies were realized for the effect of temperature using the Van ’t Hoff equation (Equation (6)):

(6) $$lnK_{c}=\frac{∆S^{0}}{R}-\frac{∆H^{0}}{RT}$$

where ∆*S*^0^ is entropy change (J·mol^−1^·K^−1^), R is the gas ideal constant (8.314 J·mol·K^−1^), ∆*H*^0^ is the enthalpy change (kJ·mol^−1^), T is the absolute temperature in *K*, and *K_d_* is the equilibrium constant obtained. ∆*H*^0^ and ∆*S*^0^ were obtained from the slope and intercept of plot LnK vs. 1/T (K^−1^). The standard Gibbs free energy (Δ*G*^0^)  was evaluated by Equation (7):

(7) $$∆G^{0}=-RTlnK_{c}$$

where *K_c_* (equilibrium constant) was evaluated at each temperature with the relation between the equilibrium concentration of the metal on the adsorbent (*C_B_*) and the equilibrium concentration of the metal in the solution (*C_A_*) (Equation (8)) [26].

(8) $$K_{c}=\frac{C_{B}}{C_{A}}$$

#### 2.2.4. Simulated Wastewater

Simulated wastewater from the battery industry used in this study was prepared according to Vergil et al., 2017 [27]. Water was doped with: Pb, 4.5 mg·L^−1^; Mn, 0.1 mg·L^−1^; Ni, 0.097 mg·L^−1^; Cu, 0.083 mg·L^−1^; Cr, 0.070 mg·L^−1^; Zn, 0.029 mg·L^−1^; Ag, 0.002 mg·L^−1^; As, 0.003 mg·L^−1^; Cd, 0.003 mg·L^−1^; and Sn, 0.014 mg·L^−1^. All metals were selected in nitrate form or in standard solution with 2% of HNO_3_ to prevent formation of precipitates.

## 3. Results and Discussions

### 3.1. Characterization of the Hybrid Membrane

#### 3.1.1. Scanning Electronic Microscopy (SEM)

**Figure 1:**
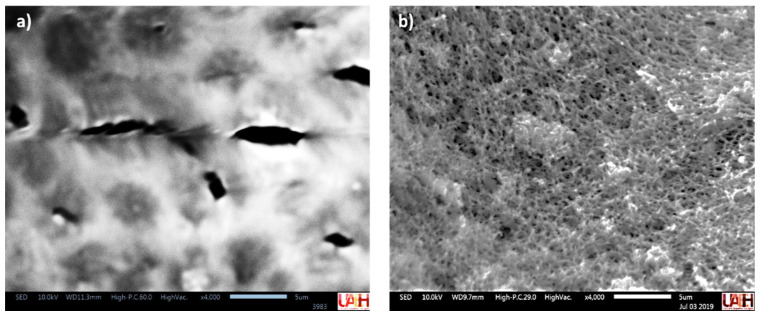
SEM microphotographs of membranes. (**a**) CA, (**b**) CA/ZnO.

SEM is a traditional technique for analyzing the surface of different materials. The characterization of the hybrid membrane by SEM is shown in Figure 1. The CA membrane presented a surface with pores of 3.9 μm (average of five measures) (Figure 1a), while the CA/ZnO (Figure 1b) showed that the addition of ZnO particles produced the formation of smaller pores such as on a sponge surface, increasing the porosity of the membrane. According to the distribution of pore size, it was an asymmetric membrane [28].

#### 3.1.2. Thermogravimetric Analysis (TGA)

**Figure 2:**
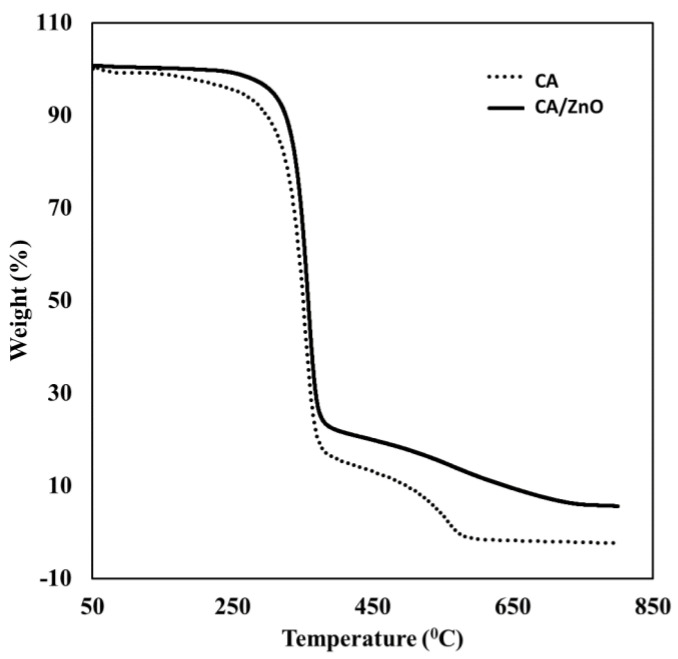
Thermogravimetric analysis of CA and CA/ZnO membranes.

The presence of ZnO and the thermal stability of the membrane were investigated by TGA. Figure 2 shows the main state of degradation; for the CA, the first loss region can be attributed to the water molecular present in the membrane. The second loss starting at ~370 °C corresponds to the degradation of cellulose acetate chains, and the third phase is due to the carbonization of the polymer. This loss was less in the CA/ZnO due to the stability provided for the particles of ZnO [29,30].

#### 3.1.3. Fourier-Transform Infrared Spectroscopy (FTIR)

**Figure 3:**
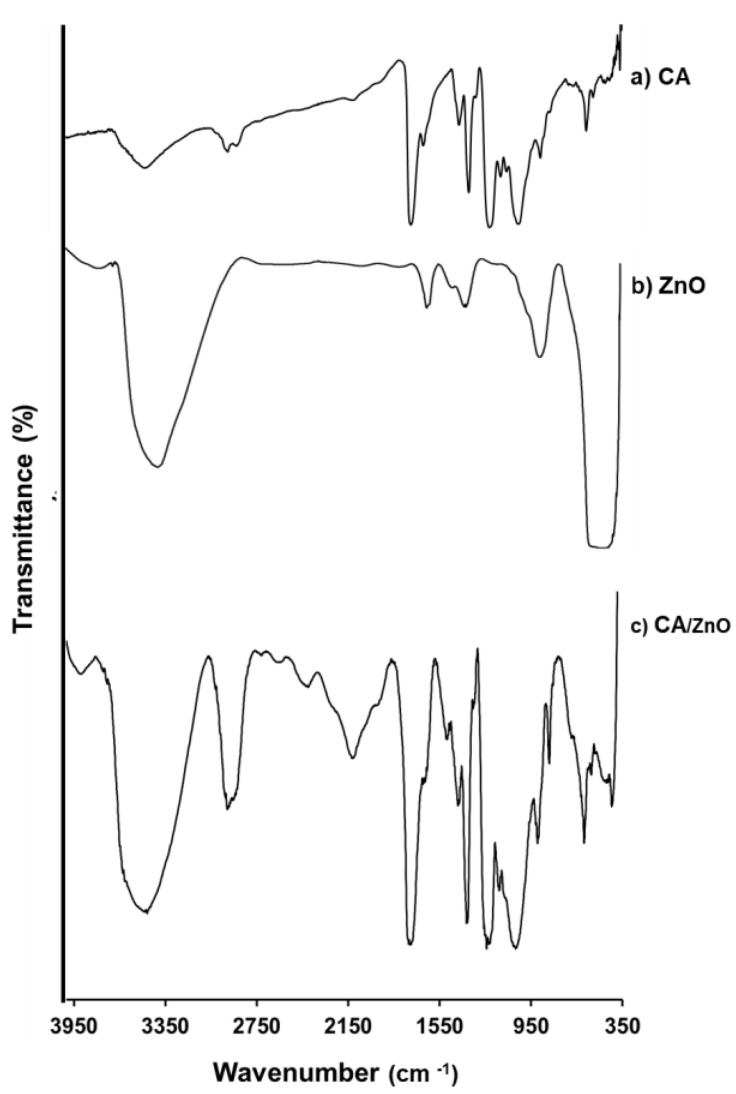
FTIR spectrum. (**a**) Cellulose acetate, (**b**) ZnO particle, (**c**) hybrid membrane of cellulose acetate and ZnO particles.

The IR spectrum of cellulose acetate showed an OH stretching vibration at 3500 cm^−1^, the CH symmetric stretching vibration of CH_2_ at 2960 cm^−1^, stretching vibration of C–O at 1756 cm^−1^, CH_2_ vibration at 1430 cm^−1^, and C–CH at 1370 cm^−1^. The characteristic peaks of cellulose are around 1000 cm^−1^. The band near 1167 cm^−1^ was due to the asymmetric stretching C–O–C 1351 cm^−1^ vibrations of CH_2_ in the cellulose (Figure 3a) [31].

In the ZnO infrared spectrum (Figure 3b), a vibration band at 450 to 550 is characteristic of a Zn–O bond; a stretching band at 3434 cm^−1^ and a bending band at 1330 to 1670 cm^−1^ can indicate a hydroxide residue [32].

On the other hand, the infrared spectrum of CA/ZnO (Figure 3c) showed an increase in the band of the Zn–O bond (450–550 cm^−1^) and the characteristics of cellulose acetate. This confirms the presence of NP in the membrane.

#### 3.1.4. Porosity and Hydrophobicity

**Figure 4:**
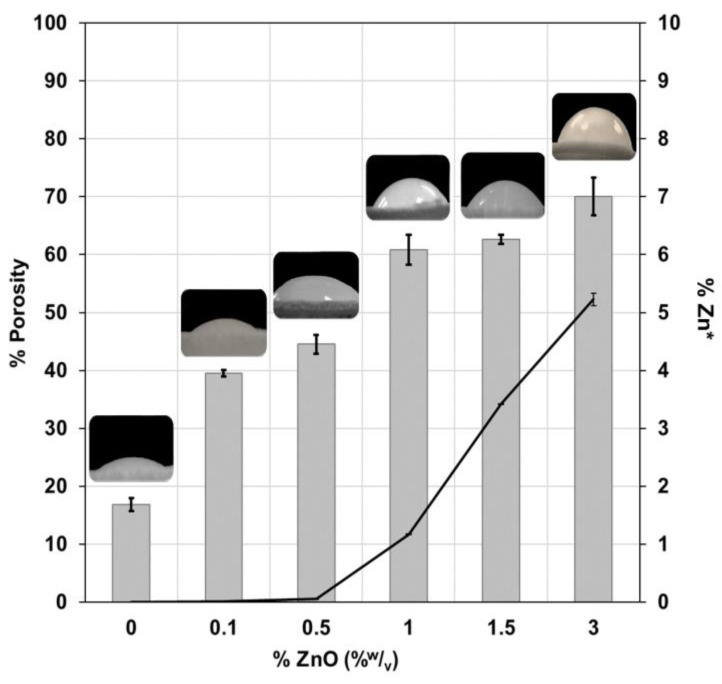
Characterization of the membrane. Percentage of porosity of CA/ZnO hybrid membrane with different ZnO concentrations (bars). Experimental conditions: 10 mL of deionized water; contact time 24 h; 2 cm^2^ of CA/ZnO prepared with 3% of NP in polymeric solution. Amount of zinc present in the membrane determined by EDS (* percentage of weight of zinc, line). Contact angle.

To verify the increase in the porosity in the membrane due to the presence of ZnO, different amounts of NP in the polymeric solution (between 0 and 3% of ZnO) were evaluated. Figure 4 shows that the percentage of porosity of CA/ZnO increased with the concentration of ZnO present in the membrane probably due to the nonsolvent concentration gradient rate difference it induced in the polymeric solution due to the increase in the viscosity of the casting solution, allowing the formation of pores [33].

On the other hand, Figure 4 also shows that there was a greater diffusion of water in the structure membrane which increased the amount of ZnO (increased the contact angle). This indicates that the presence of more NP in the CA/ZnO makes the membrane more hydrophobic; this is favorable for the antifouling ability and the water flux [34,35], and the hydrophobicity depends on ZnO/H_2_O interactions [36]. Added to this, an increase in ZnO particles in the polymer solution allows an increase in the percentage of zinc in the hybrid membrane, according to the results of energy-dispersive X-ray spectroscopy. This suggests the formation of more active sites, which facilitate the extraction process.

### 3.2. Amount of ZnO Nanoparticles

As mentioned above, oxide particles are used to give selectivity to a hybrid membrane; for this reason, the effect of the amount of NP on the CA/ZnO was evaluated in an interval of 0 to 3%. An addition of nanoparticles had a positive effect on adsorption capacity (Table 1); this may have come from the availability of active sites on the surface of CA/ZnO caused by the complexations between the lead and the oxygen of ZnO present in the surface of the membrane [37,38]. It is important to say that 3% is the maximum amount of ZnO that can be added; this was the amount used in further experiments.

### 3.3. Evaluation pH in Aqueous Solution

Different values of pH from 1 to 6 were evaluated as this permitted the facilitation of the adsorption process. Table 2 shows that, with a low pH value, the extraction of lead decreased due to the competence of protons and the Pb(II) with electrons of oxygen present in the ZnO (active sites), while, at pH 6, there was the presence of a hydroxocomplex [39]. On the other hand, at pH 5, the surface had more negative groups for the complexation of metal; for this reason, this was the value used for further experiments [40].

### 3.4. Adsorption Isotherms

Adsorption capacity between Pb(II) and ZnO/CA was analyzed by Langmuir and Freundlich models to describe the distribution between the analyte and adsorbent (see Section 2.2.3). The parameters in Table 3 suggest that the Langmuir adsorption isotherm model was the most suitable (R^2^ = 0.992 in comparison to Freundlich, which was R^2^ = 0.980), so that the energy levels were the same in all the active sites, and a homogeneous surface of adsorption without a lateral interaction between adsorbed molecules (formation of monolayer) [41,42]. The separation factor showed a favorable adsorption (R_L_ < 1), while Q_MAX_ was 15.55 mg·g^−1^, comparable to the other materials of this type used for the removal of lead (Table 4). In all the cases, the high chi-square values showed a satisfactory fit for the experimental data.

### 3.5. Thermodynamic Study

The thermodynamic parameters can be used to determine the type or nature of adsorption (see Section 2.2.3). In this case, Table 3 shows that ∆G^0^ was −29.73 to −21.52 KJ·mol^−1^, which corresponded to the physical adsorption enhanced by the chemical effect [48]. On the other hand, the ∆G^0^ negative value indicates that the adsorption of lead onto CA/ZnO was thermodynamically favorable and spontaneous, while a positive value of ∆H^0^ implies an endothermic and monolayer adsorption [49]. This result is congruent with the Langmuir isotherm model.

### 3.6. Simulated Wastewater

Adsorption of lead with CA/ZnO was evaluated from simulated battery industrial effluent and compared with an ideal system (without interferences). The percentage of lead removed was the same, in both cases greater than 97%, indicating that the other metals do not interfere with the removal of lead when employing a hybrid membrane of CA/ZnO.

## 4. Conclusions

A hybrid membrane of cellulose acetate with particles of ZnO for the removal of lead was easily developed. The characterization of CA/ZnO was realized by SEM, TGA, and IR. These techniques showed that the immobilization of ZnO in the membrane improves some properties such as stability, porosity, and hydrophobicity. The best conditions of sorption of CA/ZnO for lead were found to be: 3% of NP in the polymeric solution for the elaboration of the membrane and a solution of 10 mg·L^−1^ of lead pH = 5. The adsorption of lead was fitted with Langmuir and Freundlich isotherm models. The Langmuir model described the process as homogeneous sorption in a monolayer. The maximum capacity determined was 15.55 mg·g^−1^, comparable to similar materials. Thermodynamic parameters showed a favorable process.

The results suggest that CA/ZnO can be used for the removal of lead in the wastewater of the battery industry. The evaluation of this type of water does not show changes with respect to the ideal system, so this type of material is a great alternative, having the advantage of being a green and easy method.

All this suggests the potential of CA/ZnO for effective wastewater treatment due to its stability, chemical resistance, ion exchange capacity, and microbial effect. Hybrid membranes are a promising, low-cost strategy for eliminating pollutants present in water on an industrial scale, and there is the possibility of combining them with different separation processes.

## Figures and Tables

**Table 1:** Effect of amount of ZnO nanoparticles in the polymeric solution.

Amount of ZnO Particles (% *^W^*/*_v_*)	Percentage of Adsorption $(\%A_{Pb}$) *
0.0	27.47 (17.41)
0.1	45.18 (9.70)
0.5	65.05 (8.83)
1.0	81.02 (1.13)
1.5	82.83 (5.34)
3.0	84.81 (1.32)

* %CV in parenthesis.

**Table 2:** Effect of amount of ZnO nanoparticles’ pH solution.

pH	Percentage of Adsorption $(\%A_{Pb}$) *
1	27.47 (17.41)
2	45.18 (9.70)
3	65.05 (8.83)
4	81.02 (1.13)
5	84.81 (1.32)
6	82.83 (5.34)

* %CV in parenthesis. Experimental conditions: 10 mg·L^−1^ of Pb(II) in aqueous solution; CA/ZnO prepared with 3% of NP in polymeric solution. Contact time 4 h.

**Table 3:** Isotherm parameters obtained by linear methods for adsorption of Pb(II) onto ZnO/CA and thermodynamics parameters.

Langmuir					Freundlich			
Q_o_ (mg/g)	K_L_ (L/ mg)	R_L_	R^2^	χ^2^	K_F_ (mg/g)	n_o_	R^2^	χ^2^
15.55	0.035	0.06–0.97	0.992	4.304	1.77	2.76	0.980	0.819
**Thermodynamic parameters**								
Temperature (K)		∆G^0^ (KJ mol^−1^)			∆H^0^ (KJ mol^−1^)		∆S^0^ (KJ mol^−1^K^−1^)	
291		−21.52			119.41		0.48	
298		−24.66						
308		−29.73						

Experimental conditions: 10 mg L^−1^ of Pb(II) in aqueous solution at pH = 5; CA/ZnO prepared with 3% of NP in polymeric solution.

**Table 4:** Comparison of maximum capacity adsorption of lead by different adsorbents.

Adsorbent	Qmax (mg·g^−1^)	Reference
Iron oxide nanoparticles immobilized in sand	2.087	[43]
Ultrafiltration membrane of polysulfone with hydrous ferric oxide	13.20	[44]
Hybrid membrane of cellulose acetate with zinc oxide	15.55	This work
Aluminum oxide	17.50	[45]
Iron-oxide-coated bentonite	22.20	[46]
Polyacrylonitrile with metal–organic framework (MOF-808) membrane	23.98	[47]
Magnesium-oxide-coated bentonite	31.86	[46]

## Data Availability

Not applicable.
